# Isolation and identification of *SiCOL5*, which is involved in photoperiod response, based on the quantitative trait locus mapping of *Setaria italica*

**DOI:** 10.3389/fpls.2022.969604

**Published:** 2022-09-20

**Authors:** Fei-fei Li, Jia-hong Niu, Xiao Yu, Qing-hua Kong, Run-feng Wang, Ling Qin, Er-ying Chen, Yan-bing Yang, Zhen-yu Liu, Li-na Lang, Hua-wen Zhang, Hai-lian Wang, Yan-an Guan

**Affiliations:** ^1^Featured Crops Engineering Laboratory of Shandong Province, National Engineering Research Center of Wheat and Maize, Shandong Technology Innovation Center of Wheat, Crop Research Institute, Shandong Academy of Agricultural Sciences, Jinan, China; ^2^College of Life Science, Shandong Normal University, Jinan, China; ^3^Shandong Seed Administration Station, Jinan, China

**Keywords:** foxtail millet, high-density linkage map, photoperiod sensitivity, QTL mapping, SiCOL5

## Abstract

Foxtail millet (*Setaria italica*) is a versatile grain and fodder crop grown in arid and semi-arid regions. It is an especially important crop for combating malnutrition in certain poverty-stricken areas of the world. Photoperiod sensitivity is a major constraint to the distribution and utilization of foxtail millet germplasm resources. Foxtail millet may be suitable as a model species for studying the photoperiod sensitivity of C_4_ crops. However, the genetic basis of the photoperiod response of foxtail millet remains poorly studied. To detect the genetic basis of photoperiod sensitivity-related traits, a recombinant inbred line (RIL) population consisting of 313 lines derived from a cross between the spring-sown cultivar “Longgu 3” and the summer-sown cultivar “Canggu 3” was established. The RIL population was genotyped using whole-genome re-sequencing and was phenotyped in four environments. A high-density genetic linkage map was constructed with an average distance between adjacent markers of 0.69 cM. A total of 21 quantitative trait loci (QTLs) were identified by composite interval mapping, and 116 candidate genes were predicted according to gene annotations and variations between parents, among which three genes were considered important candidate genes by the integration and overall consideration of the results from gene annotation, SNP and indel analysis, *cis*-element analysis, and the expression pattern of different genes in different varieties, which have different photoperiod sensitivities. A putative candidate gene, *SiCOL5*, was isolated based on QTL mapping analysis. The expression of *SiCOL5* was sensitive to photoperiod and was regulated by biological rhythm-related genes. Function analysis suggested that *SiCOL5* positively regulated flowering time. Yeast two-hybrid and bimolecular fluorescence complementation assays showed that SiCOL5 was capable of interacting with SiNF-YA1 in the nucleus.

## Introduction

Foxtail millet (*Setaria italica*) is among the first domesticated grain crops and is a versatile grain and fodder crop cultivated in arid and semi-arid regions. It is an important crop for combating malnutrition in areas with chronic poverty. Foxtail millet is a short-day crop and is sensitive to photoperiod. Therefore, the plant has extremely limited ecological adaptability, and photoperiod sensitivity is a major constraint to the exchange of foxtail millet germplasm resources. It also accounts for the scarcity of broadly adaptable foxtail millet cultivars in commercial production, which limits further development and capacity to strengthen the competitiveness of the millet seed industry. Therefore, improvement of the adaptability of foxtail millet cultivars is important to expand and strengthen the millet seed industry (Jia and Diao, 2022).

Photoperiod is an important factor that regulates the flowering period of crops to ensure that flowering occurs at the correct time, which determines the regional adaptability of crops. Research on photoperiod regulation of flowering has mainly focused on model plants, such as *Arabidopsis* (*Arabidopsis thaliana*) and rice (*Oryza sativa*). *Arabidopsis* is a classic model plant, and its photoperiodic regulation of flowering has been studied in considerable detail, which can be summarized as follows: When there is an optical signal, *PHY* and *CRY* receive the optical signal, and through integration of the *ELF3* and *ZTL* genes, the signal is transmitted to the core oscillator, which then outputs the signal to *GI*, *FKF1*, *CO*, and other downstream genes. CO promotes flowering by inducing *FT*, *LFY*, and *SOCl* (Anwer et al., 2018; Sim et al., 2019). As a typical gramineous crop, research on rice provides an important reference for other gramineous crops. Many crucial genes in the photoperiodic control of flowering have been detected in rice by quantitative trait locus (QTL) mapping, such as *Hd3a*, *Hd1*, *Ehd1*, and *Ghd7* (Yano, 2000; Kazuyuki et al., 2004; Tsuji et al., 2008; Xue et al., 2009); two pathways promote flowering in a short-day environment, namely, *OsGI–Hd1–Hd3a* and *OsGI–Ehd1–Hd3a*. Similarly, two pathways restrain flowering in a long-day environment, namely, *OsELF3–OsGI–Hd1–Hd3a* and *PhyA–Ghd7–Ehd1–Hd3a*, and one pathway promotes flowering in a long-day environment, namely, *OsMADS50–Ehd1–RFT1* (Lee et al., 2004). However, rice is a C_3_ crop and is only distantly related to C_4_ gramineous crops, such as corn (*Zea mays*), sorghum (*Sorghum bicolor*), sugarcane (*Saccharum* spp.), and foxtail millet.

Among C_4_ crops, most studies of photoperiod sensitivity have focused on maize and sorghum. Several important genes associated with photoperiod, such as *SbEhd1*, *SbCO*, *SbPRR37*, and *SbGHD7*, have been cloned from sorghum (Murphy et al., 2011; Yang et al., 2014; Klein et al., 2015). Photoperiod response in maize has been studied extensively using QTL mapping (Chardon et al., 2004; Wang et al., 2008; Coles et al., 2010). The results of QTL mapping in maize indicate that the bin 10.04 region of chromosome 10 had the greatest additive effects and was the most stable QTL. The research reported that this maize chromosomal region includes at least three photoperiod-sensitive genes: *ZmCCT*, *ZmPIF3*, and *ZmCCA1*. In recent years, the functions of several major genes have been studied. In maize, *ZCN8* and *Conz1* are considered to be homologs of *FT* and CO, respectively, in *Arabidopsis* (Miller et al., 2008). The gene *ZmCCT* is a homolog of *Ghd7* in rice. Jin et al. (2018) speculated that *ZmCOL3* might inhibit flowering through positive regulation of *ZmCCT* expression or inhibition of the biological clock. Cheng et al. (2018) showed that *ZmCCT9* inhibited flowering by suppressing *ZCN8* expression. Su et al. (2018) speculated that ZmNF–YA3 may form trimers with CO-like and FPF1 and bind to the promoter of *ZmFT-like12* to promote its expression and thus accelerate the flowering transition. Thus, several photoperiod-sensitive genes have been identified and cloned in sorghum and maize, and a number of genes have been functionally studied. However, given the large genomes and complex regulatory mechanisms of maize and sorghum, it is difficult to elucidate the mechanism that controls photoperiod sensitivity response in C_4_ crops.

Foxtail millet is an ideal model species for the study of photoperiod response in C_4_ crops owing to its high inbreeding rate, C_4_ photosynthesis, small diploid genome, and abundant genetic resources with different sensitivities to photoperiod. Studies on photoperiod regulation in foxtail millet are scarce compared with those on maize and sorghum. To date, a small number of studies have been conducted on QTL mapping for photoperiod sensitivity-related traits in foxtail millet (Jia et al., 2013; Mauro-Herrera et al., 2013; Doust et al., 2017; Ni et al., 2017; Zhang et al., 2017). Xie (2012) showed that QTLs for photoperiod sensitivity-related traits in a short-day environment are located on chromosome 4 of foxtail millet, and QTLs for such traits in a long-day environment are located on chromosomes 3 and 9 of foxtail millet. Jia et al. (2013) identified 512 loci associated with 47 agronomic traits from a genome-wide association analysis of foxtail millet. However, the genetic basis of the photoperiod response of foxtail millet remains poorly understood.

Nevertheless, a small number of QTLs and genes associated with photoperiod sensitivity response have been reported in foxtail millet. Therefore, in this study, a high-density genetic map was constructed by whole-genome re-sequencing, the QTLs were mapped with short intervals, and a crucial candidate gene was cloned and identified. The findings provide valuable genetic information for innovation of the germplasm resources of foxtail millet and novel gene resources to accelerate the breeding of broadly adapted foxtail millet cultivars.

## Materials and methods

### Plant materials and field trials in different environments

A recombinant inbred line (RIL) mapping population consisting of 313 F_8_ individuals was derived from a cross between the foxtail millet cultivars “Canggu 3” and “Longgu 3.” “Longgu 3” is a cultivar from northwestern China sensitive to photoperiod, whereas “Canggu 3” is from North China insensitive to photoperiod. The RIL population was generated using the single seed descent method.

Field trials for evaluation of the phenotypic performance of the RIL population were conducted in four environments (location/years or months) in China. The four environments comprised two locations: Jinan Experimental Station in Shandong Province and the Sanya Experimental Station in Hainan Province. The Jinan Experimental Station was adopted for field trials in May–September 2017, June–October 2017, and June–October 2018, and the Sanya Experimental Station was adopted for field trials in November–January 2017. These location/year or month combinations were designated 2017CQ, 2017XQ, 2018Q, and 2017HQ, respectively. A total of two replications were included in all environments using a randomized complete block design. Every line was grown in a two-row plot of length 500 cm, with a spacing of 3 cm between individuals and 50 cm between rows.

### Measurement and analysis of phenotypic data for photoperiod sensitivity-related traits

For QTL analysis of photoperiod sensitivity-related traits, previous research has shown that heading date (HD), panicle length (PL), and panicle weight (PW) are suitable indicator traits for photoperiod sensitivity evaluation of foxtail millet (Jia et al., 2018). Therefore, HD, PL, and PW were recorded in each environment. In all environments, the phenotyping evaluation standard was identical. HD was defined as the period from the day of seedling emergence to the day when more than half of the individuals in a plot attained heading. PL was defined as the distance from the base to the apex of the main panicle. In total, five plants were randomly selected for evaluation of each phenotypic trait. The mean of the two replicates (blocks) for each phenotypic trait was used for QTL analysis.

### Phenotype statistical analysis

The heritability, frequency distributions, and coefficient of variance (CV) were analyzed using SPSS Statistics version 17.0. The broad-sense heritability (*H*^2^) of the photoperiod sensitivity-related traits was calculated in accordance with the method of Knapp et al. (1985) using the formula *H*^2^ = δ_*g*_^2^/(δ_*g*_^2^ + δ_*ge*_^2^/*e* + δ^2^/*e* × *r*), where δ_*ge*_^2^ is the genotype × environment interaction, δ_*g*_^2^ is the genetic variance, δ^2^ is the error variance, *r* is the number of replications per environment, and *e* is the number of environments.

### High-density genetic map construction

To construct DNA libraries for the RIL population, fresh leaves were sampled from each individual plant and immediately stored at −80°C until use. Genomic DNA was extracted using the Plant Genomic DNA Kit (TIANGEN, Beijing, China) in accordance with the manufacturer’s protocol.

A re-sequencing strategy was adopted for high-density single-nucleotide polymorphism (SNP) development and RIL population genotyping. The genome of each RIL was re-sequenced on an Illumina HiSeq 2500 platform, and the raw data were analyzed by Illumina CASAVA 2.17 (Levy and Myers, 2016). Low-quality reads were filtered, and the remaining data were used for SNP calling. The clean reads were aligned to the reference genome of foxtail millet using the Burrows–Wheeler Aligner (Mckenna et al., 2010). The alignments were further analyzed and converted to BAM files for SNP calling and genotyping with SAMtools software (Li and Durbin, 2009). The SNPs were annotated using the Genome Analysis Toolkit (Mckenna et al., 2010), Picard, and SnpEff (Depristo et al., 2011; Cingolani et al., 2012).

A sliding window method was adopted to calculate the ratio of SNP alleles from “Longgu 3” and “Canggu 3” (Huang et al., 2009). The genotype data were scanned with 15 SNPs per window and a step size of 1 SNP. Consecutive SNPs with the same genotype were gathered into blocks. The locus between two different genotype blocks was determined to be the recombination breakpoint. A bin marker was defined as a consecutive 10-kb interval in which a recombination event was absent. A high-density genetic linage map was constructed by mapping the bin markers on the nine foxtail millet chromosomes using HighMap (Liu et al., 2014).

### Quantitative trait locus identification and candidate gene prediction

For mapping the QTLs associated with the photoperiod sensitivity-related traits, composition interval mapping and the “R/qtl” package were used. A logarithm of the odds (LOD) score greater than 2.5 was chosen as the threshold for declaration of the presence of a putative QTL in a particular region. The confidence interval was the region in which the LOD value intersected the threshold line.

The genes located in the confidence intervals were annotated with BLASTX (Altschul et al., 1997) by searching NR, COG, GO, and SwissProt 9 databases. Based on amino acid similarity, candidate genes located in all QTL intervals showing homology to genes related to photoperiod response in *Arabidopsis* or rice were identified, and putative *cis*-acting elements of the candidate genes were analyzed. Candidate genes located in the major QTL intervals with variations in the CDS region between two parents were identified.

Based on the RNA-seq data obtained,^1^ the relative expression levels of all candidate genes were assessed in Yugu1 and A10. Yugu1 is insensitive to photoperiod, while A10 is sensitive to photoperiod. Furthermore, previous research suggested that the response of A10 to photoperiod was varying in different growth stages, that is, at the 1- to 3-leaf stage, the photoperiod response is the most sensitive, and then the sensitive effect decreased obviously at the 4- to 11-leaf stage, whereas when A10 reached the 12-leaf stage, the photoperiod response becomes the most insensitive. Therefore, we selected RNA-seq data from the samples on the first leaf at the seedling stage (the sensitive phase of photoperiod response) and on the flag leaf at the booting stage (the insensitive phase of photoperiod response) for expression pattern analysis.

Furthermore, several important candidate genes were selected for expression analysis by qRT-PCR in 20 millet varieties having dissimilar photoperiod sensitivities, and the information of millet varieties is given in Supplementary Table 5. The most prospective candidate genes were shortlisted by the integration and overall consideration of the results from gene annotation, SNP and indel analysis, *cis*-element analysis, and expression analysis of candidate genes.

### Cloning and function analysis of the candidate gene *SiCOL5*

The *SiCOL5* gene sequence was downloaded from the Phytozome database. The *SiCOL5* cDNA was amplified, and then the PCR products were purified and inserted into the pCAMBIA1300 vector. The recombination vector was introduced into *Agrobacterium tumefaciens* strain GV3101 and then transformed into *Arabidopsis* by using the floral dip method. Transgenic plants were selected by culture on plates supplemented with hygromycin and then were transferred to soil. The transgenic plants used for phenotypic analysis were T_3_ lines.

For expression characterization analysis of *SiCOL5*, fresh intact roots, stems, flag leaves, leaf sheaths, and panicles were collected. For analysis of circadian expression, fresh leaf samples were collected at 3-h intervals over a 48-h period from plants growing under short-days (light 9 h, dark 15 h) or long-days (light 15 h, dark 9 h). All samples were collected with three biological replicates. The sampled leaves were immediately frozen in liquid N_2_ and stored at −80°C until use. The expression pattern of *SiCOL5* was analyzed by quantitative real-time PCR (qRT-PCR). The relative expression level of *SiCOL5* was calculated using the 2^–Δ^ ^Δ^ ^Ct^ method.

To analyze the function and regulatory network of *SiCOL5*, yeast two-hybrid (Y2H) assays were conducted. The fusion expression vectors pGADT7-SiCOL5 and PGBKT7-SiNF-YA1 were constructed, and then the fusion expression vector pGADT7-SiCOL5 + PGBKT7-SiNF-YA1, the positive vector pGADT7-T + PGBKT7-53, and negative control vector pGADT7-T + PGBKT7-lam were transformed separately into yeast (*Saccharomyces cerevisiae*) strain AH109 by using the polyethylene glycol/lithium acetate method. The yeast strains were cultured on the SD-T-L and SD-T-L-H-A plates for assessment of interaction between SiCOL5 and SiNF-YA1 in yeast.

To analyze the subcellular location of the SiCOL5 protein and further examine interaction between SiCOL5 and SiNF-YA1, the fusion expression vectors p35S-SiCOL5-GFP, pSAT1-SiCOL5-nEYFP, and pSAT1-SiNF-YA1-cEYFP were generated by using a homologous recombination method. Protoplasts were isolated from rice leaf cells and were transformed with the fusion expression vector p35S-SiCOL5-GFP + p35S-OsGhd7-GFP, the control vector GFP + p35S-OsGhd7-GFP, or the pSAT1-SiCOL5-nEYFP + pSAT1-SiNF-YA1-cEYFP vector. The transformed protoplasts were incubated for 16–18 h in the dark. Signals from the green fluorescent protein (GFP) and the yellow fluorescent protein (YFP) were observed using a confocal laser scanning microscope (FLV1200, Olympus, Tokyo, Japan).

## Results

### Phenotype identification and analysis

**FIGURE 1:**
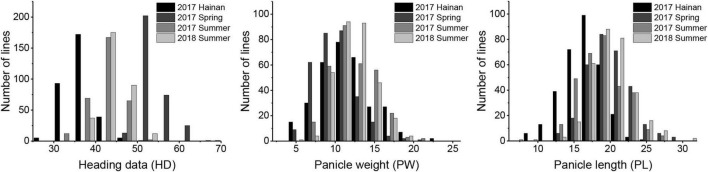
Frequency distributions for heading date, panicle length, and panicle weight in the foxtail millet recombinant inbred line (RIL) population.

“Longgu 3” and “Canggu 3” differed significantly in photoperiod sensitivity-related traits. Considerable phenotypic variation was observed in HD, PL, and PW in the RIL population across the four environments. Phenotypic values of HD, PW, and PL showed a normal distribution curve, which indicated that HD, PW, and PL are governed by multiple genes (Figure 1). Statistical analysis of the data for each phenotypic trait is presented in Table 1 and Supplementary Table 1. The CV for the three traits ranged from 6.72 to 29.54%. The broad-sense heritability of the three traits ranged from 87.48 to 93.06%. These results indicated that the three traits were largely under heritable genetic control.

**TABLE 1:** Summary of phenotype data for quantitative traits of the foxtail millet recombinant inbred line (RIL) population in four environments.

Trait	Environments	Parents		RILs							
		M	P	Max.	Min.	Mean	SD	Skewness	Kurtosis	CV (%)	*H*^2^ (%)
Panicle length (cm)	17CQ	19.77	21.25	29.50	12.30	19.73	2.94	0.35	0.24	14.12	88.99
	17XQ	16.75	20.45	26.78	11.10	18.71	3.00	0.33	−0.36	16.03	
	17HQ	17.05	9.64	24.30	8.30	16.35	2.64	−0.30	0.31	16.14	
	18XQ	22.60	25.67	31.75	7.58	19.93	2.92	0.10	1.97	14.66	
Panicle weight per plant (g)	17CQ	12.18	6.52	21.37	4.45	10.08	2.66	0.78	1.30	26.38	87.48
	17XQ	12.40	9.63	21.31	6.25	12.03	2.72	0.37	−0.15	22.62	
	17HQ	12.85	5.14	23.83	4.00	11.47	3.39	0.29	0.46	29.54	
	18XQ	13.80	10.36	19.62	4.79	12.21	2.38	0.32	0.17	19.49	
Heading date (days)	17CQ	53	50	68	47	53.45	3.59	1.15	0.97	6.72	93.06
	17XQ	42	42	51	33	41.56	3.55	−0.11	−0.24	8.54	
	17HQ	39	26	53	26	36.51	3.68	0.51	1.14	10.09	
	18XQ	43	41	69	36	43.13	3.57	1.45	8.01	8.29	

M: “Canggu 3”; P: “Longgu 3.” *H*^2^: broad-sense heritability. CV, coefficient of variation.

### Basic characteristics of the genetic map

A total of 2,076 bins were determined, which suggested that many recombination events had occurred in the RIL population (Supplementary Figure 1). A high-density genetic linage map was constructed by mapping the 2,076 bin markers onto the nine foxtail millet chromosomes. The total genetic distance of the constructed linkage map was 1,417.97 cM, and the average distance between adjacent markers was 0.69 cM. The map contained 107,396 SNP loci and 2,076 bin markers. The average distance between bin markers ranged from 0.43 to 1.33 cM on a single chromosome (Table 2). Collinearity analysis showed that the linkage map was highly accurate, and Spearman correlation coefficients were greater than 0.99.

**TABLE 2:** Description of the basic characteristics of the genetic map for foxtail millet.

Linkage group ID^a^	Bin marker	Total distance (cM)^b^	Average distance (cM)
Chr1	325	179.90	0.56
Chr2	293	129.91	0.44
Chr3	155	140.38	0.91
Chr4	214	172.11	0.81
Chr5	200	183.38	0.92
Chr6	142	188.08	1.33
Chr7	418	179.83	0.43
Chr8	142	127.85	0.91
Chr9	187	116.53	0.63
Total	2076	1417.97	0.69

^a^Chr: linkage group ID on each chromosome. ^b^Total distance is the total genetic distance of a linkage group.

### Quantitative trait loci identification and prediction of candidate genes

**FIGURE 2:**
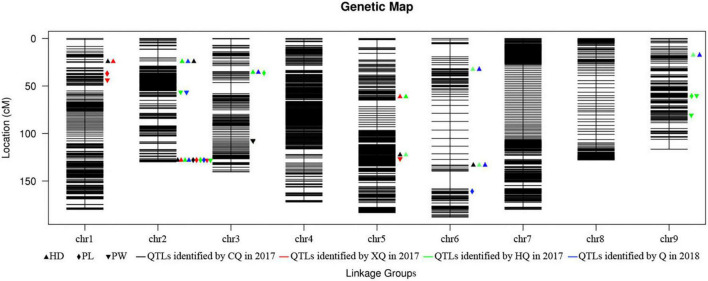
High-density genetic map and summary of the identified QTLs.

A total of 21 QTLs for HD, PW, and PL were identified (Figure 2). The phenotypic variation explained (PVE) value ranged from 1.64% (qPW5) to 69.92% (qHD1) within a trait (Table 3). Further analysis showed that 12 QTLs were detected in more than two environments, which were viewed as stable QTLs (shown in red or blue in Supplementary Figure 2). The retained QTLs that were detected in a single environment were considered to be unique QTLs (shown in green in Supplementary Figure 2).

**TABLE 3:** Quantitative trait locus (QTL) mapping of three quality traits in the four environments.

QTL	Chr^a^	Marker	Genetic position (cM)			Physical position (Mb)			LOD^b^	PVE (%)^c^	ADD^d^	Environment
			Start	End	Distance	Start	End	Distance				
qHD1	1	Block544-545	18.93	18.93	0.00	1821152	2021933	0.20	5.18	69.92	−3.97	17CQ
		Block544-545	18.93	18.93	0.00	1821152	2021933	0.20	2.79	43.01	−3.08	17XQ
qHD2-1	2	Block5837-5914	20.32	23.62	3.29	2849742	3018544	0.17	3.09	0.75	0.41	17CQ
		Block5762-8152	11.14	30.82	19.68	2015203	8716474	6.70	4.86	6.32	1.21	17HQ
		Block5914	23.62	23.62	0.00	2994608	3018444	0.02	5.74	1.32	0.54	18Q
qHD2-2	2	Block14217	129.27	129.27	0.00	49169304	49182261	0.01	23.79	15.01	−1.84	17CQ
		Block14217	129.27	129.27	0.00	49169304	49182261	0.01	24.38	14.48	−1.79	17XQ
		Block14218	129.91	129.91	0.00	49183322	49200481	0.02	15.60	13.40	1.76	17HQ
		Block14205-14214	128.44	128.73	0.29	49095683	49166432	0.07	26.76	15.09	−1.84	18Q
qHD3	3	Block17835-17407	34.90	35.23	0.32	14213058	15842258	1.63	6.40	24.61	−2.39	17HQ
		Block17835-17407	34.90	35.23	0.32	14213058	15842258	1.63	4.03	10.19	−1.51	18Q
qHD5-1	5	Block29236-29591	50.20	64.94	14.75	7699919	9843267	2.14	5.68	2.43	0.73	17XQ
		Block29236-29591	50.20	64.94	14.75	7699919	9843267	2.14	5.08	5.13	1.09	17HQ
qHD5-2	5	Block29853-30143	111.56	125.13	13.58	13960422	24201025	10.24	2.67	0.63	0.38	17CQ
		Block29853-30211	111.56	134.24	22.69	13960422	24939701	10.98	3.25	4.96	1.07	17HQ
qHD6-1	6	Block33509	31.38	31.38	0.00	2042285	2052917	0.01	4.78	5.32	1.11	17HQ
		Block33116-34243	25.39	39.52	14.13	1388998	3280815	1.89	3.71	0.61	0.37	18Q
qHD6-2	6	Block36389-36421	137.85	138.34	0.48	27488651	28190802	0.70	4.50	5.50	−1.11	17CQ
		Block36389-36454	137.85	138.66	0.80	27488651	28255863	0.77	4.21	17.86	−2.04	17HQ
		Block36389-36392	137.85	138.01	0.16	27488651	27561418	0.07	2.83	3.09	−0.83	18Q
qHD9	9	Block60743-60773	20.06	20.28	0.22	5875509	5986760	0.11	2.66	3.97	0.96	17HQ
		Block60623-61055	13.33	24.19	10.87	4644491	8508650	3.86	3.54	1.48	0.56	18Q
qPL1	1	Block640-647	36.70	37.19	0.48	3496523	3770570	0.27	2.97	5.62	−0.94	17XQ
qPL2	2	Block14199-14214	128.02	128.73	0.70	48989024	49166432	0.18	9.98	8.18	−1.12	17CQ
		Block14217	129.27	129.27	0.00	49169304	49182261	0.01	26.00	19.94	−1.77	17XQ
		Block14195-14205	127.64	128.44	0.80	48946140	49116719	0.17	8.06	7.36	0.94	17HQ
		Block14205-14214	128.44	128.73	0.29	49095683	49166432	0.07	15.40	12.02	−1.33	18Q
qPL3	3	Block17614-17938	37.34	37.34	0.00	14964445	17317622	2.35	3.49	32.99	−1.99	17HQ
qPL6	6	Block37109-37120	176.34	177.04	0.71	32605531	32710929	0.11	4.43	4.00	−0.77	18Q
qPL9	9	Block61962-62010	57.49	57.97	0.48	40159357	40514420	0.36	3.44	3.97	0.69	17HQ
qPW1	1	Block890-993	48.95	49.27	0.32	5552939	6153865	0.60	2.71	2.75	−0.60	17XQ
qPW2-1	2	Block12177-12193	56.87	57.09	0.22	28277872	28383815	0.11	2.84	3.02	0.78	17HQ
		Block12177-12196	56.87	57.32	0.45	28277872	28420257	0.14	3.80	2.60	0.51	18Q
qPW2-2	2	Block14217-14218	129.27	129.91	0.64	49169304	49200481	0.03	6.20	4.50	−0.77	17XQ
		Block14217-14218	129.27	129.91	0.64	49169304	49200481	0.03	6.24	6.67	1.15	17HQ
qPW3	3	Block19556-19559	122.62	123.07	0.45	41416024	41855285	0.44	3.06	2.68	0.58	17CQ
qPW5	5	Block30267-30250	136.36	136.81	0.45	25373723	25484206	0.11	4.27	1.64	0.46	17XQ
qPW9-1	9	Block61951-61985	57.49	57.49	0.00	40159357	40321677	0.16	2.87	3.48	0.83	17HQ
qPW9-2	9	Block63401-63509	79.05	80.99	1.94	49748436	51702279	1.95	3.01	3.31	0.81	17HQ

^a^Chr: chromosome. ^b^LOD, logarithm of the odds. ^c^PVE, phenotypic variance explained. ^d^ADD, additive effect.

Among the QTLs, five robust and major ones (PVE > 10%) were selected, but qHD2-2 and qPL2 were at the same locus (Supplementary Table 2). The QTL qHD1 had the largest effect and had 69.92 and 43.01% of the phenotypic variation in 2017CQ and 2017XQ, respectively. This region contained 20 annotated genes. The QTL qHD2-2 had a strong influence on HD and had more than 10% of the phenotypic variation in all four environments. It is noteworthy that qHD2-2 was not only significant but also pleiotropic, and this region was associated with HD, PL, and PW. In a QTL analysis of the three traits combined, a crucial region was located adjacent to the Block14205–14218, corresponding to a physical distance of approximately 0.01 Mb (2017CQ and 2017XQ) to 0.07 Mb (2018Q) (Table 3), and this region contained 21 genes. The detailed list of the candidate genes of qHD1 and qHD2-2 can be found in Supplementary Table 3.

In all QTL intervals, a total of 2,803 genes were annotated by using the GO, COG, NR, and SwissProt databases. Overall, 19 photoperiod sensitivity-related candidate genes were predicted based on the gene annotations (Supplementary Table 4). The candidate genes were assigned to nine gene families, including the CCT family, NF-Y transcription factor family, and MADS-box family (Supplementary Figure 3). Analysis of putative *cis*-elements in the candidate genes indicated that most of the candidate genes had light-responsive elements (Supplementary Figure 4).

In the major QTL interval, a total of 270 genes were annotated, among which 97 genes had variations in the CDS region (Supplementary Table 2). In the qHD1 interval, Seita.1G021700 and Seita.1G021200, coding for uncharacterized protein, had non-synonymous mutation. In the qHD2-2 interval, Seita.2G445400 and Seita.2G444200 had frame shift variation, the function of which has not been characterized in millet. The variant of Seita.2G444300 between parents can be found in many regions, including CDS, upstream, intron, and downstream. Seita.2G444300 is a two-component response regulator-like PRR37 gene according to the Nr annotation, so we regarded Seita.2G444300 as the most probable candidate gene in the qHD2-2 interval. In the qHD3 interval, Seita.3G205400 and Seita.3G195900 contained premature stop codons; nevertheless, they code for uncharacterized protein. There were eight genes with frame shift variation. In the qHD6-2 interval, Seita.6G155600 and Seita.6G15860 contained a premature stop codon, and Seita.6G155600 is a cytochrome P450 71A1-like protein. Seita.6G155500 and Seita.6G155500 have frame shift variation. The detailed variation information between parents is given in Table 4.

**TABLE 4:** Variation analysis between parents of the candidate genes in major QTLs.

QTL	Gene ID	Nr annotation	Position	Ref	P	M	Variation type
qHD1	Seita.1G021700	Uncharacterized protein	1904650	G	A	C	Non-synonymous coding
	Seita.1G021200	Uncharacterized protein	1831278	C	C	C	Non-synonymous coding
qHD2-2	Seita.2G445400	Uncharacterized protein	49185509	TAG	T,TAG	TAG	Frame shift
	Seita.2G444200	Uncharacterized protein	49117637	AT	A	AT	Frame shift
	Seita.2G443600	Glycosyltransferase	49098978	C	CGCTGCT	C	Codon insertion
	Seita.2G443700	Uncharacterized protein	49100803	GAAC	GAAC	G	Codon insertion
	Seita.2G445100	Uncharacterized protein	49172292	G	C	G	Non-synonymous coding
	Seita.2G445200	Uncharacterized protein	49175179	C	T	C	Non-synonymous coding
	Seita.2G444700	Hypothetical protein SETIT_031465mg	49148770	G	A	G	Non-synonymous coding
	Seita.2G444900	Telomeric repeat-binding factor 2	49160124	C	A	C	Non-synonymous coding
	Seita.2G444600	Protein NETWORKED 2D	49142634	T	C	T	Non-synonymous coding
	Seita.2G444400	Probable serine/threonine-protein kinase PBL7	49138518	C	A	C	Non-synonymous coding
	Seita.2G443700	Uncharacterized protein LOC101756159	49100311	A	G	A	Non-synonymous coding
	Seita.2G443500	Phosphoinositide phospholipase C 2 isoform X2	49095673	G	C	G	Non-synonymous coding
	Seita.2G444800	Hypothetical protein SETIT_033421mg, partial	49150047	A	T	A	Non-synonymous coding
	Seita.2G444300	Like PRR37	49129307	G	A	G	Non-synonymous coding
qHD3	Seita.3G205400	Hypothetical protein SETIT_004917mg, partial	15836737	G	A	G	Stop gain
	Seita.3G195900	Uncharacterized protein	14896536	G	A	G	Stop gain
	Seita.3G189400	22.3 kDa class VI heat shock protein	14352409	CT	C,CT	CT	Frame shift
	Seita.3G197900	Hypothetical protein PAHAL_C02536	15000739	GT	G	GT	Frame shift
	Seita.3G195100	Uncharacterized protein	14839859	CCA	C	CCA	Frame shift
	Seita.3G199600	Uncharacterized protein	15101694	T	TA	T	Frame shift
	Seita.3G187700	H/ACA ribonucle protein complex NAF1	14228108	G	GCC,G	G	Frame shift
	Seita.3G198500	PREDICTED: ADP-ribosylation factor 2 isoform X1	15030744	CCTCT	C	CCTCT	Frame shift
	Seita.3G198600	Lipid transfer protein GPI-anchored 2-like isoform	15038941	C	CT	C	Frame shift
	Seita.3G195600	Myb family transcription factor PHL7	14870485	T	TA	T	Frame shift
	Seita.3G188400	Universal stress protein PHOS32	14266230	C	C,C	C	Codon deletion
	Seita.3G197000	Uncharacterized protein	14948561	GAGA	G	GAGA	Codon deletion
	Seita.3G203900	Probable WRKY transcription factor 63	15637813	T	TAGAGCATCC	T	Codon insertion
	Seita.3G204200	LRR receptor-like serine/threonine-protein kinase	15699729	G	GGGA,G	G	Codon insertion
	Seita.3G195300	Transcription factor TGAL1	14850912	A	G	A	Non-synonymous coding
	Seita.3G190100	Hypothetical protein SETIT_025028mg	14374820	G	A	G	Non-synonymous coding
	Seita.3G198900	NADH-cytochrome b5 reductase 1	15057250	T	C	T	Non-synonymous coding
	Seita.3G195000	Disease resistance protein RGA2 OS	14841648	G	A	G	Non-synonymous coding
qHD6-2	Seita.6G155600	Cytochrome P450 71A1-like	27559673	G	G	A	Stop gain
	Seita.6G158600	Uncharacterized protein	28097208	T	T	A	Stop gain
	Seita.6G155500	Uncharacterized protein	27556232	G	G	GCA	Frame shift
	Seita.6G159200	Uncharacterized protein	28214989	T	T	TC	Frame shift
	Seita.6G158900	Myb family transcription factor PHL8	28185819	T	T	TGACGCG	Codon insertion
	Seita.6G155500	Uncharacterized protein	27553999	A	A	G	Non-synonymous coding
	Seita.6G158900	Uncharacterized protein	28185596	C	C	T	Non-synonymous coding
	Seita.6G157000	Uncharacterized protein	27794643	G	A	G	Non-synonymous coding
	Seita.6G156500	Uncharacterized protein	27695570	A	A	G	Non-synonymous coding
	Seita.6G159400	Uncharacterized protein	28223049	T	T	C	Non-synonymous coding

The expression patterns of all candidate genes were analyzed using RNA-seq data from Yugu1 and A10. The results showed that Seita.1G065300, Seita.2G444300, Seita.5G178100, Seita.9G468100, Seita.3G195200, Seita.6G156100, Seita3G1956100, and Seita.3G198500 had different expression patterns at the seedling stage between Yugu1 and A10. The expression levels of Seita.1G065300, Seita.2G444300, Seita.5G178100, Seita.9G468100, Seita.3G195200, and Seita.6G156100 were higher in A10 than those in Yugu1; on the contrary, the expression levels of Seita.3G195600 and Seita.3G198500 were lower in A10 than those in Yugu1. At the booting stage, the expression differences of Seita.1G065300, Seita.2G444300, Seita.3G198500, and Seita.6G156100 between Yugu1 and A10 were attenuated or insignificant; the expression profiles of these genes agreed with the result that the 12-leaf stage is insensitive to photoperiod response (Supplementary Figure 5).

**FIGURE 3:**
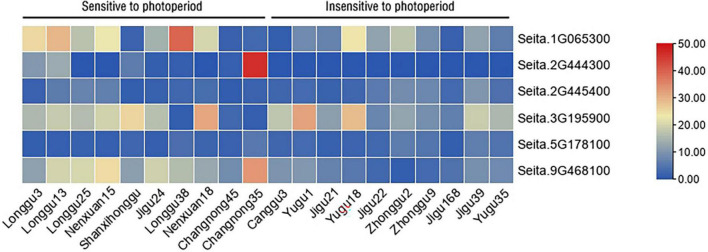
Expression analysis of important candidate genes in different millet varieties having different photoperiod sensitivities. Different colors in the heatmap represent gene transcript values.

Furthermore, six candidate genes, namely, Seita.1G065300, Seita.2G444300, Seita.9G468100, Seita.5G178100, Seita.2G445400, and Seita.3G195900, were selected for expression analysis by qRT-PCR in 20 millet varieties which had dissimilar photoperiod sensitivities. The qRT-PCR results showed that Seita.1G065300, Seita.2G444300, and Seita.9G468100 had different expression patterns in two categories of varieties having different photoperiod sensitivities. The expression patterns of Seita.1G065300, Seita.2G444300, and Seita.9G468100 were similar, and they all had higher expression levels in millet varieties sensitive to photoperiod. This result agreed with the RNA-seq data. It is interesting to note that the expression of Seita.2G444300 was not detected in all selected millet varieties insensitive to photoperiod but only in four varieties sensitive to photoperiod. The expression level of Seita.2G445400 and Seita.5G178100 was low in all selected millet varieties. The expression of Seita.3G195900 is irregular in the two categories of millet varieties which had different photoperiod sensitivities (Figure 3).

In summary, among the candidate genes, Seita.1G065300, Seita.2G444300, and Seita.9G468100 were selected as important candidate genes for photoperiod response by the integration and overall consideration of the results from gene annotation, SNP and indel analysis, *cis*-element analysis, and the expression pattern of different genes in different varieties having different photoperiod sensitivities.

### Cloning and preliminary function analysis of the candidate gene *SiCOL5*

**FIGURE 4:**
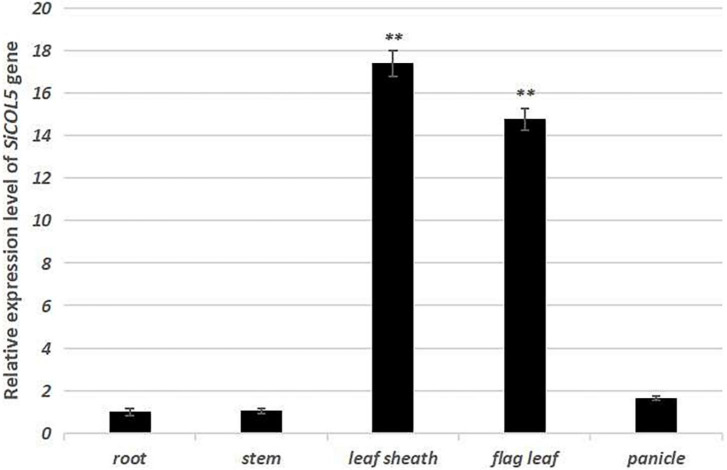
Expression patterns of the candidate gene *SiCOL5* in various tissues of foxtail millet. ^∗∗^*p* < 0.01.

**FIGURE 5:**
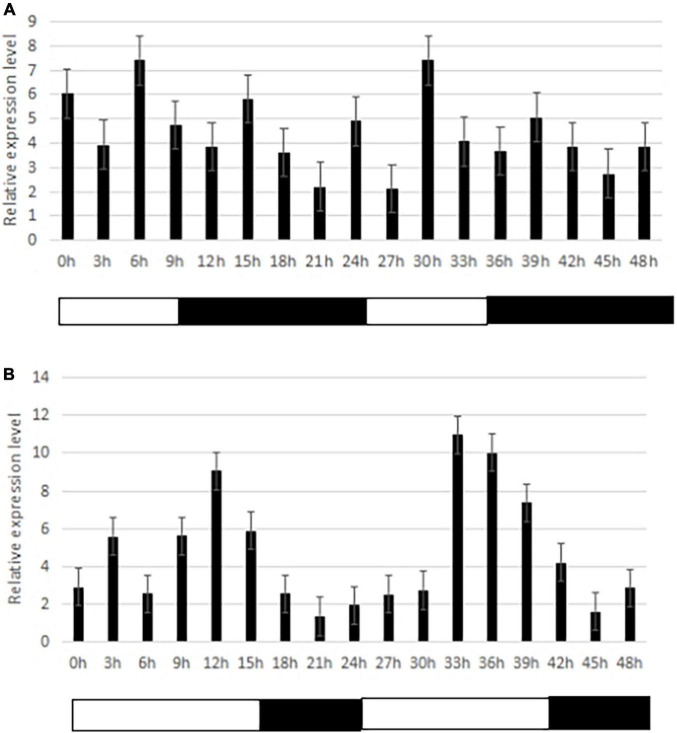
Circadian expression of the candidate gene *SiCOL5* under different photoperiod treatments. **(A)** Short-days; **(B)** long-days. Black bars represent the dark period, and white bars represent the light period.

**FIGURE 6:**
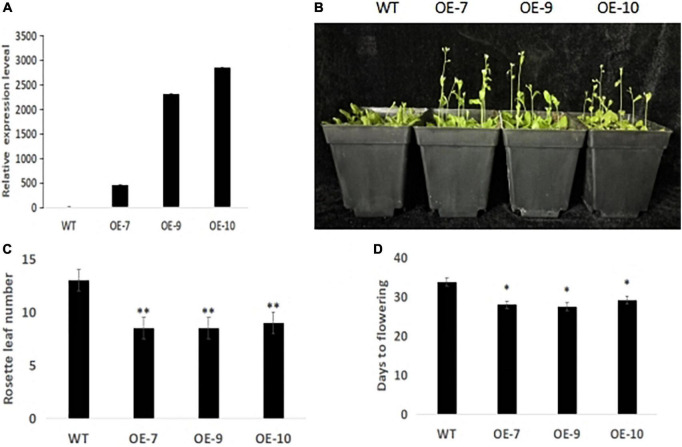
*SiCOL5* positively regulates flowering time in *Arabidopsis*. **(A)** Identification of *SiCOL5* overexpression plants by qRT-PCR. **(B–D)** Phenotype **(B)**, rosette leaf number **(C)**, and days to flowering **(D)** of *SiCOL5* overexpression plants compared with wild-type plants. **p < 0.01, *p < 0.05.

One important candidate gene was cloned and was designated *SiCOL5*. The gene encoded a 386-amino acid protein and belonged to the CCT family. The qRT-PCR analysis indicated that *SiCOL5* transcripts were more abundant in the flag leaf and leaf sheath than in other tissues (Figure 4), which was consistent with the public expression data. It also suggested that *SiCOL5* may perform a crucial function in the leaf. Furthermore, the expression of *SiCOL5* showed a circadian pattern in short-day and long-day environments (Figure 5). However, the circadian expression of *SiCOL5* differed under the two photoperiods. These results suggested that *SiCOL5* was sensitive to photoperiod and was regulated by biological rhythms. The *SiCOL5* gene was ectopically expressed in *Arabidopsis* to investigate the function of *SiCOL5*. The transgenic *Arabidopsis* lines overexpressing *SiCOL5* produced fewer leaves and bloomed earlier (Figure 6) than wild-type *Arabidopsis*. The results suggested that *SiCOL5* positively regulated the flowering time in *Arabidopsis*.

**FIGURE 7:**
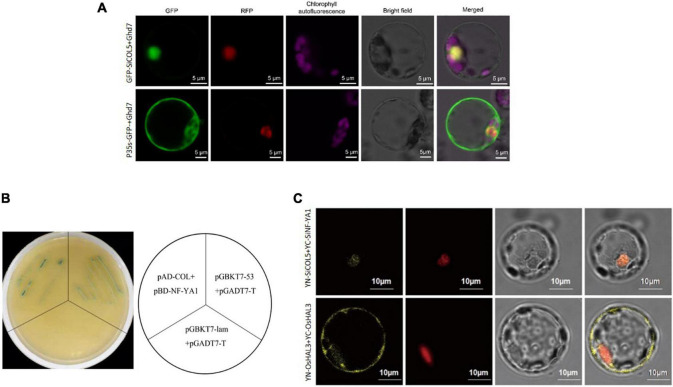
Interaction between SiCOL5 and SiNF-YA1 in the nucleus. **(A)** Subcellular localization of SiCOL5. **(B,C)** Interaction between SiCOL5 and SiNF-YA1 in a yeast two-hybrid assay **(B)** and bimolecular fluorescence complementation assay **(C)**. Scale bar = 10 μm.

Subcellular localization analysis showed that SiCOL5 protein was localized in the nucleus (Figure 7A). A Y2H assay showed that SiCOL5 was capable of interacting with SiNF-YA1 in yeast (Figure 7B). This was confirmed by performing a bimolecular fluorescence complementation (BiFC) assay in rice protoplasts. These results indicated that SiCOL5 interacted with SiNF-YA1 in the nucleus (Figure 7C).

## Discussion

Cultivars that show broad adaptability in the commercial production and breeding of foxtail millet are scarce, which strongly limits further development and strengthening of the competitive power of the millet seed industry. Therefore, it is important to expand and strengthen the millet seed sector by improving the adaptability of foxtail millet cultivars (Jia and Diao, 2022). However, the genetic basis of the photoperiod sensitivity response of foxtail millet and the main effect genes involved in regulating the photoperiod sensitivity response remain elusive. Mapping and identification of the major genes that regulate the photoperiod sensitivity response are vital to achieve rapid and directional genetic improvement of cultivar adaptability.

The resolution of genetic maps has an important impact on the accuracy of QTL mapping. Compared with previously reported genetic maps of foxtail millet (Zhang et al., 2012; Qie et al., 2014), such as a random fragment length polymorphism-based map (Wang et al., 1998), a simple sequence repeat linkage map (Jia et al., 2009), and a genetic map constructed with 992 SNPs (Bennetzen et al., 2012), a greater number of markers were used for construction of the present map, and the marker density and resolution of the present genetic map were higher. Therefore, a greater number of recombination events could be detected. Based on the high-density genetic map, narrowing of the regions between markers and prediction of candidate genes could be achieved more accurately.

In the present study, 21 QTLs were detected, but a majority of those were novel, and only a small number of those were consistent with previously detected QTLs. This may be due to differences in the environments or populations used between studies. Xie (2012) detected QTLs for photoperiod sensitivity-related traits on chromosome 4 of foxtail millet grown under short-days, and on chromosomes 3 and 9 of foxtail millet grown under long-days. However, we failed to detect corresponding QTLs on chromosome 4. The reason may be that Xie used flowering date as the phenotypic trait, whereas we selected HD as a photoperiod sensitivity-related trait. However, only highly heritable and stable QTLs can be used for marker-assisted selection. In this study, qHD1 and qHD2-2 were stable QTLs and had more than 10% of phenotypic variation. It is notable that Jia et al. (2013) also detected genomic regions showing strong association signals for HD on chromosomes 1 and 2. Therefore, we suggest that qHD1 and qHD2-2 could be considered priority candidates for marker-assisted selective breeding.

In the present study, 116 candidate genes were predicted. Among the candidate genes, Seita.2G085600 and Seita.2G444300 also were mapped by genome-wide association by Jia et al. (2013). Seita.2G444300 (SiPRR37) was reported to be involved in photoperiod sensitivity in millet; therefore, the results verified the QTL mapping results of this study. In addition, most candidate genes identified in this study were novel genes, such as the candidate genes in the major QTL intervals. The QTL qHD1 had the largest effect and had 69.92 and 43.01% of phenotypic variation in 2017CQ and 2017XQ, respectively. In the qHD1 interval, Seita.1G021700 and Seita.1G021200 had non-synonymous mutation, and they were uncharacterized protein. In qHD2-2, Seita.2G445400 and Seita.2G444200 had frame shift variation, the function of which also has remained uncharacterized by now in millet. Therefore, the uncharacterized genes in the major QTL intervals that had important variants between parents should be prospective, which could be used for foxtail millet breeding.

We cloned *SiCOL5* following expression analysis of the candidate genes (Figure 3 and Supplementary Figure 5). Xilei et al. (2020) analyzed advances in research on molecular mechanisms of the photoperiod regulation of plant flowering and the *CCT* gene family. They suggested that the CCT family plays a critical role in photoperiod regulation of the flowering pathway. LinLin et al. (2021) reported that a significant genome-wide association signal (position: 31456761 bp) was detected on chromosome 1, and in this region, only one CCT family gene (*SiTOC1*) was identified. Further analysis identified many haplotypic variations of *SiTOC1*, but the REC and CCT domains of *SiTOC1* were conserved in foxtail millet accessions (LinLin et al., 2021). These results also suggested that the CCT domain plays a key role in photoperiod regulation of the flowering pathway.

In this study, some QTLs were pleiotropic or showed tight linkage with other QTLs, such as qHD2-2. This finding suggested that the regions with the pleiotropic QTLs may be hot spots that control photoperiod sensitivity response-related traits. Functional identification of the genes in the hot spot regions is important for breeding. In addition, many separate QTLs were mapped that controlled HD, PL, or PW, rather than two or three traits, which suggests that each trait should have a relatively independent genetic pathway. Therefore, fine mapping of the QTL is needed for QTL cloning. The present results provide an important basis for further fine mapping of QTLs and contribute to elucidation of the molecular mechanisms of photoperiod responses in foxtail millet.

In the current study, 12 QTLs for HD, PL, and PW were stable QTLs that were detected in multiple environments; thus, the QTLs were insensitive to different environments. There may be two reasons for this: the QTLs may have important effects on phenotypes, or the QTLs may have been overlooked *via* current statistical methods, although they exert a large genotype–environment interaction effect.

## Conclusion

A high-density linkage map was constructed for foxtail millet based on large-scale development of SNPs and bin markers by re-sequencing. Using this high-density map, three photoperiod sensitivity-related phenotypic traits were mapped, and 21 QTLs were identified in four environments. In total, 116 candidate genes were predicted, and three major genes were selected. An important candidate gene, *SiCOL5*, was cloned and identified. These results provide valuable genetic information for the innovation of germplasm resources of foxtail millet and novel gene resources to accelerate the breeding of foxtail millet cultivars with broad adaptability.

## Data availability statement

The original contributions presented in the study are publicly available. This data can be found here: NCBI, PRJNA853459.

## Author contributions

F-FL and Y-AG coordinated the project, and conceived and designed the experiments. J-HN, XY, and Q-HK conducted the experiments. R-FW conducted the bioinformatics work. F-FL wrote the first draft. LQ, E-YC, and Y-BY edited the manuscript. L-NL, Z-YL, H-WZ, and H-LW contributed valuable discussions. All authors have read and approved the final manuscript version.

## Footnotes

1
http://foxtail-millet.biocloud.net/home

## Supplementary material

Supplementary Figure 1 Recombination bin map for the foxtail millet recombinant inbred line (RIL) population. The bin map comprises 2076 bin markers. Physical position is based on Yugu1 RefGen-V2.2 sequence. Red: “Longgu 3” genotype; blue: “Canggu 3” genotype; yellow: heterozygote.

Supplementary Figure 2 Summary of QTL identifications. **(A)** QTL mapping for heading date (HD). **(B)** QTL mapping for panicle length (PL). **(C)** QTL mapping for panicle weight (PW). QTLs that were expressed stably in all four environments are shown in red. QTLs that were stable in two or three environments are shown in blue. QTLs that were specifically expressed in only one environment are shown in green.

Supplementary Figure 3 Classification of candidate genes associated with photoperiod sensitivity response in foxtail millet.

Supplementary Figure 4 Putative *cis*-element analysis of the candidate genes associated with flowering. Different *cis*-elements are indicated by different colors. Light-responsive elements, low temperature-responsive elements, and gibberellin-responsive elements were analyzed.

Supplementary Figure 5 Expression profiles of the candidate genes in Yugu1 and A10. Different colors in the heatmap represent gene transcript values.

## Abbreviations

qRT-PCR: quantitative real-time PCR
WT: wild type
GFP: green fluorescent protein
QTL: quantitative trait locus
Y2H: yeast two-hybrid
BiFC: bimolecular fluorescence complementation
HD: heading date
PL: panicle length
PW: panicle weight.
