# Supplementary figures and images for: Isolation and identification of SiCOL5, which is involved in photoperiod response, based on the quantitative trait locus mapping of Setaria italica
Source: Front Plant Sci. 2022 Sep 20;13:969604. doi: 10.3389/fpls.2022.969604 (PMC9530826; doi:10.3389/fpls.2022.969604)

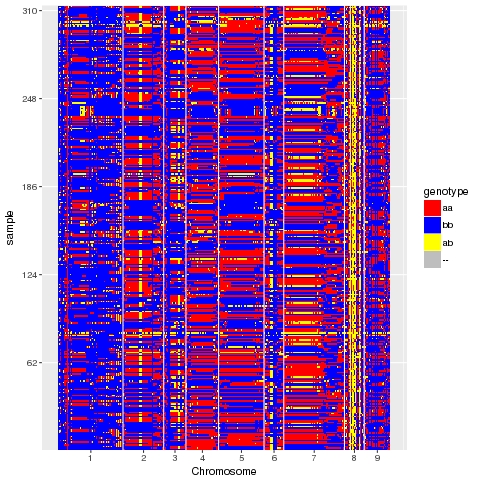

Supplement: Supplementary Figure 1 — Recombination bin map for the foxtail millet recombinant inbred line (RIL) population. The bin map comprises 2076 bin markers. Physical position is based on Yugu1 RefGen-V2.2 sequence. Red: “Longgu 3” genotype; blue: “Canggu 3” genotype; yellow: heterozygote. [file Image_1.JPEG]

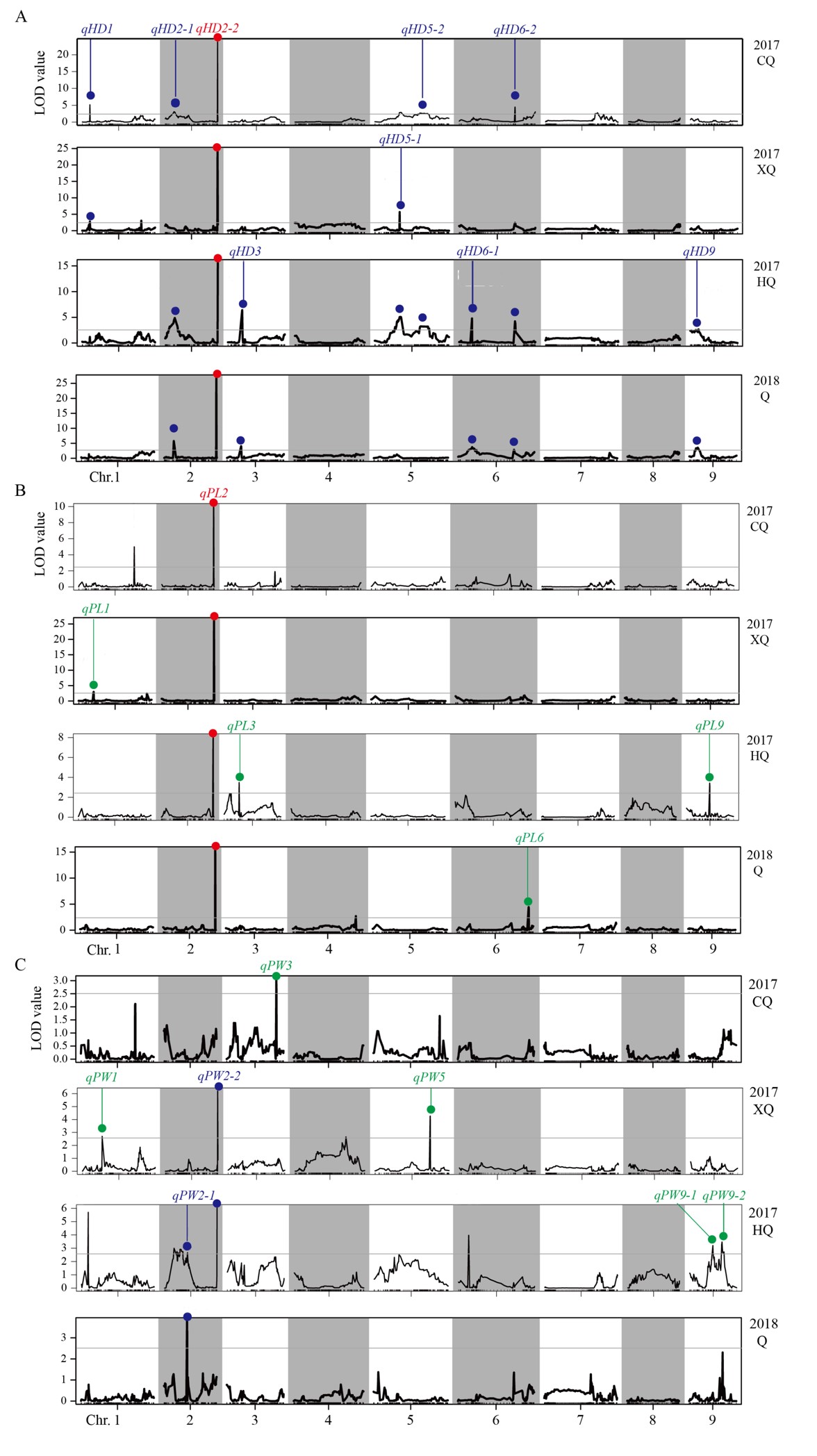

Supplement: Supplementary Figure 2 — Summary of QTL identifications. (A) QTL mapping for heading date (HD). (B) QTL mapping for panicle length (PL). (C) QTL mapping for panicle weight (PW). QTLs that were expressed stably in all four environments are shown in red. QTLs that were stable in two or three environments are shown in blue. QTLs that were specifically expressed in only one environment are shown in green. [file Image_2.JPEG]

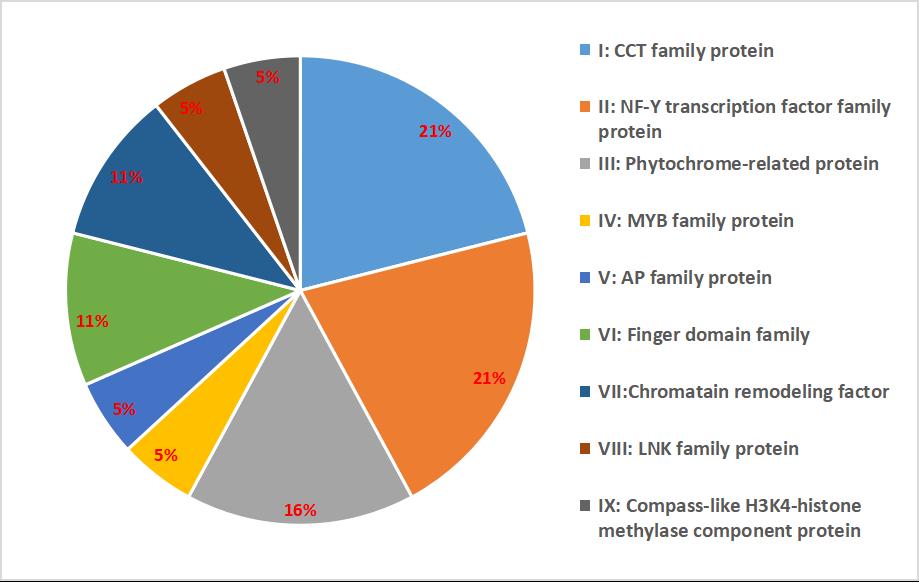

Supplement: Supplementary Figure 3 — Classification of candidate genes associated with photoperiod sensitivity response in foxtail millet. [file Image_3.JPEG]

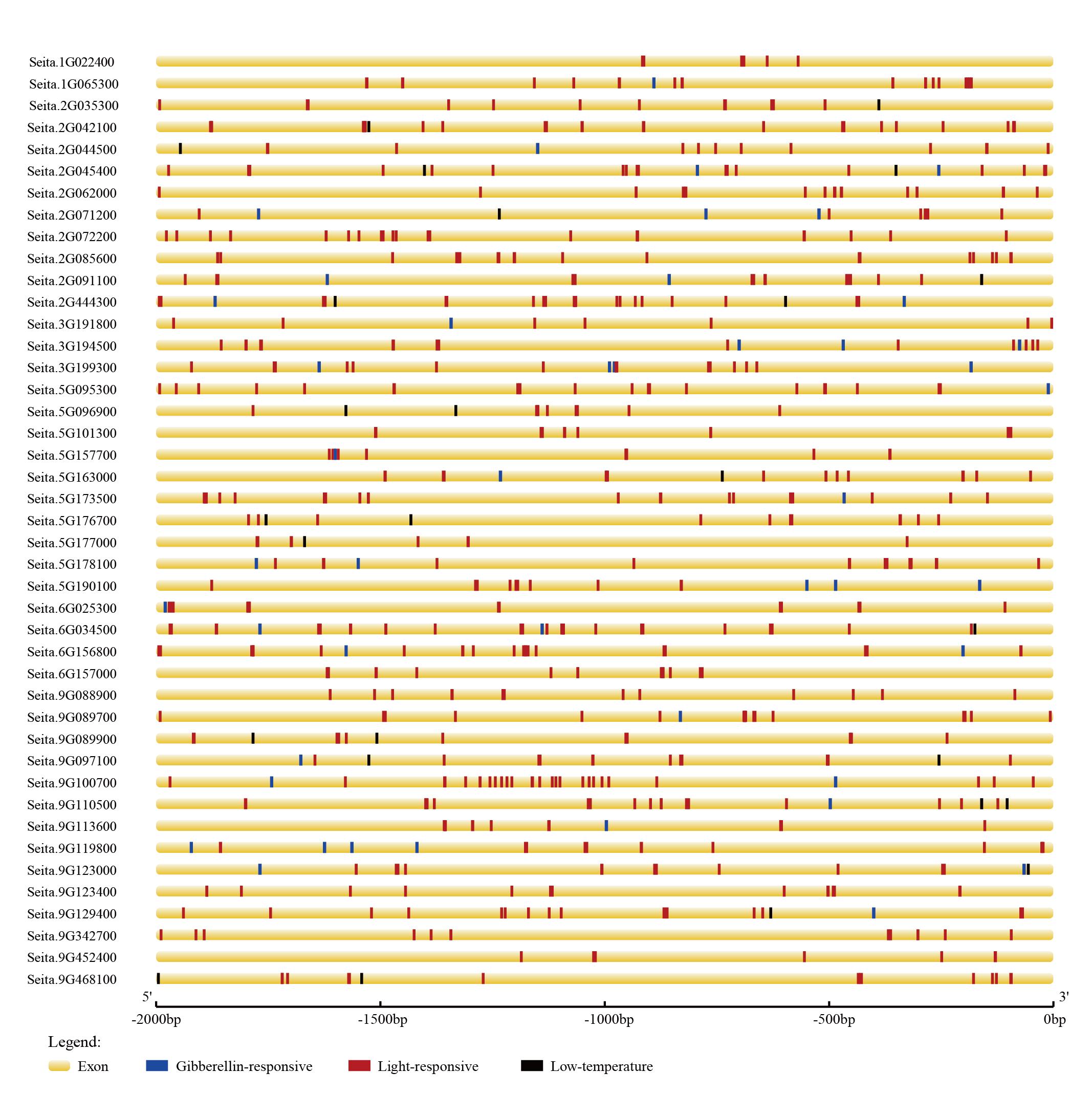

Supplement: Supplementary Figure 4 — Putative cis-element analysis of the candidate genes associated with flowering. Different cis-elements are indicated by different colors. Light-responsive elements, low temperature-responsive elements, and gibberellin-responsive elements were analyzed. [file Image_4.JPEG]

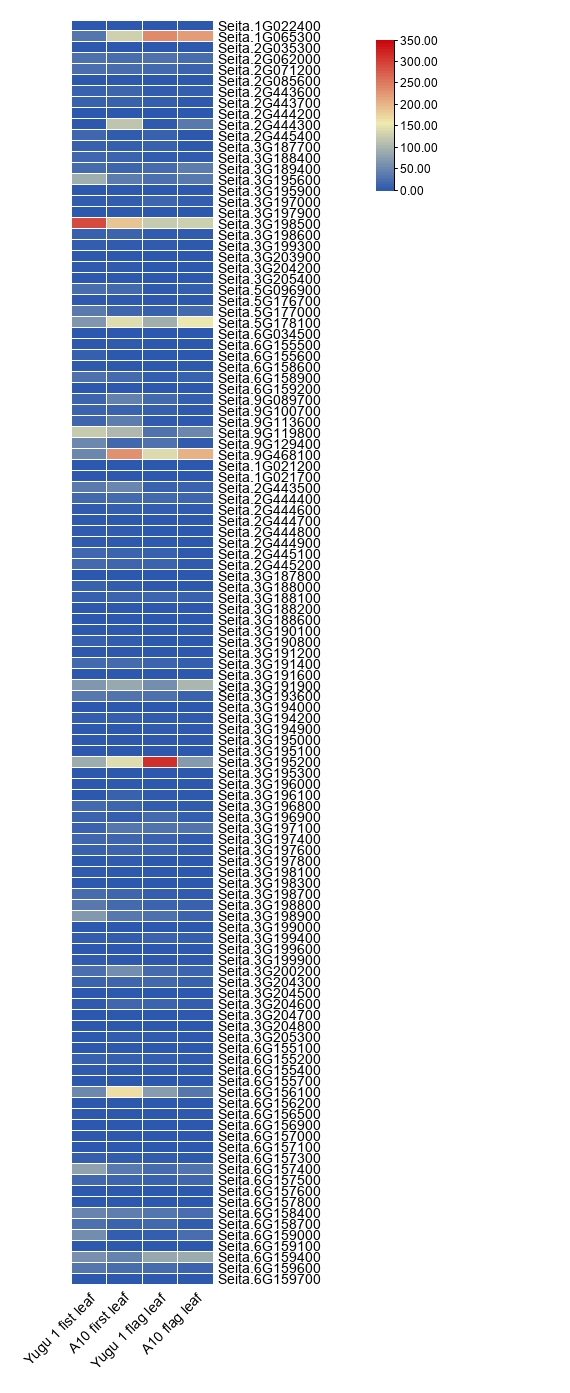

Supplement: Supplementary Figure 5 — Expression profiles of the candidate genes in Yugu1 and A10. Different colors in the heatmap represent gene transcript values. [file Image_5.JPEG]
