# Supplementary material for: Isolation and identification of SiCOL5, which is involved in photoperiod response, based on the quantitative trait locus mapping of Setaria italica
Source: Front Plant Sci. 2022 Sep 20;13:969604. doi: 10.3389/fpls.2022.969604 (PMC9530826; doi:10.3389/fpls.2022.969604)
Supplement: Supplementary file 6 [file Data_Sheet_1.docx]

**TABLE S1** Variance analysis of the three measured traits across four environments in the RIL population

| Trait | Variance | MS | F | *P* Value | H^2^(%) |
| --- | --- | --- | --- | --- | --- |
| PL | Environment  Genotype  Genotype*Environment  Error | 164.41 | 618.904 | <2E-16 | 88.99 |
|  |  | 28.2 | 10.613 | <2E-16 |  |
|  |  | 12.6 | 4.727 | <2E-16 |  |
|  |  | 2.7 |  |  |  |
| PW | Environment  Genotype  Genotype*Environment  Error | 574.5 | 1213.61 | <2E-16 | 87.48 |
|  |  | 23.5 | 49.68 | <2E-16 |  |
|  |  | 13.2 | 27.98 | <2E-16 |  |
|  |  | 0.5 |  |  |  |
| HD | Environment  Genotype  Genotype*Environment  Error | 31612 | 15472.341 | <2E-16 | 93.06 |
|  |  | 57 | 27.661 | <2E-16 |  |
|  |  | 16 | 7.654 | <2E-16 |  |
|  |  | 2 |  |  |  |

**TABLE S2**  Information of the stable and main QTL(PVE>10 )

| QTL | LOD^b^ | PVE(%)^c^ | Environment | Gene number | Number of gene had variations in CDS region |
| --- | --- | --- | --- | --- | --- |
| qHD1 | 5.18 | 69.92 | 17CQ | 20 | 2 |
|  | 2.79 | 43.01 | 17XQ |  |  |
| qHD2-2 | 23.79 | 15.01 | 17CQ | 21 | 13 |
|  | 24.38 | 14.48 | 17XQ |  |  |
|  | 15.60 | 13.40 | 17HQ |  |  |
|  | 26.76 | 15.09 | 18Q |  |  |
| qHD3 | 6.40 | 24.61 | 17HQ | 179 | 55 |
|  | 4.03 | 10.19 | 18Q |  |  |
| qHD6-2 | 4.5 | 5.5 | 17CQ | 50 | 27 |
|  | 4.21 | 17.86 | 17HQ |  |  |
|  | 2.83 | 3.09 | 18Q |  |  |
| qPL2 | 9.98 | 8.18 | 17CQ | 21 | 13 |
|  | 26.004 | 19.938 | 17XQ |  |  |
|  | 8.06 | 7.36 | 17HQ |  |  |
|  | 15.40 | 12.02 | 18Q |  |  |

**TABLE S3** The candidate gene list of the major QTL

| QTL | Marker | Candidate gene | Nr annotation |
| --- | --- | --- | --- |
| qHD1 | Block544-545 | Seita.1G022200 | peroxidase 70-like |
|  |  | Seita.1G021800 | hypothetical protein SETIT_020110mg |
|  |  | Seita.1G022400 | cationic peroxidase 1 |
|  |  | Seita.1G021400 | hypothetical protein SETIT_016359mg |
|  |  | Seita.1G021700 | uncharacterized protein |
|  |  | Seita.1G022800 | thermospermine synthase ACAULIS5 |
|  |  | Seita.1G021600 | hypothetical protein SETIT_019258mg |
|  |  | Seita.1G022100 | uncharacterized protein LOC101767640 |
|  |  | Seita.1G021200 | uncharacterized protein |
|  |  | Seita.1G022300 | peroxidase P7 |
|  |  | Seita.1G022500 | peroxidase 70 |
|  |  | Seita.1G021900 | calmodulin-binding receptor kinase CaMRLK |
|  |  | Seita.1G022900 | hypothetical protein SETIT_017097mg |
|  |  | Seita.1G021500 | uncharacterized protein LOC101755438 |
|  |  | Seita.1G021100 | hypothetical protein SETIT_019090mg |
|  |  | Seita.1G022700 | uncharacterized protein |
|  |  | Seita.1G022600 | uncharacterized protein |
|  |  | Seita.1G021300 | hydroquinone glucosyl transferase |
|  |  | Seita.1G023000 | hypothetical protein SETIT_017813mg |
|  |  | Seita.1G022000 | hypothetical protein SETIT_019139mg, partial |
| qHD2-2 | Block14205-14218 | Seita.2G444700 | hypothetical protein SETIT_031465mg |
|  |  | Seita.2G444200 | uncharacterized protein |
|  |  | Seita.2G444900 | telomeric repeat-binding factor 2 |
|  |  | Seita.2G443600 | probable glucuronosyl transferase Os07g0694400 |
|  |  | Seita.2G444600 | protein NETWORKED 2D |
|  |  | Seita.2G444400 | probable serine/threonine-protein kinase PBL7 |
|  |  | Seita.2G444100 | hypothetical protein SETIT_030030mg |
|  |  | Seita.2G443900 | arogenate dehydratase/prephenate dehydratase 2, chloroplastic |
|  |  | Seita.2G443700 | uncharacterized protein LOC101756159 |
|  |  | Seita.2G443500 | phosphoinositide phospholipase C 2 isoform X2 |
|  |  | Seita.2G444000 | L-ascorbate peroxidase 2, cytosolic |
|  |  | Seita.2G445000 | 2-oxoglutarate dehydrogenase, mitochondrial |
|  |  | Seita.2G444800 | hypothetical protein SETIT_033421mg, partial |
|  |  | Seita.2G443800 | probable sugar phosphate/phosphate translocator At3g11320 |
|  |  | Seita.2G444300 | two-component response regulator-like PRR37 |
|  |  | Seita.2G444500 | protein IN2-1 homolog B isoform X2 |
|  |  | Seita.2G445100 | nucleoporin autopeptidase |
|  |  | Seita.2G445200 | nucleoporin autopeptidase |
|  |  | Seita.2G445300 | hras-like suppressor-related protein |
|  |  | Seita.2G445400 | nucleolar preribosomal-associated protein 1 |
|  |  | Seita.2G445500 | uncharacterized protein |

**TABLE S4** The candidate genes related to photoperiod sentivity response

| QTL | Gene | Best-hit Arabidopsis name | Gene name in Arabidopsis | Best-hit rice name | Classification |
| --- | --- | --- | --- | --- | --- |
| qHD1 | Seita.1G022400 | AT5G06720 | PA2 | LOC_Os02g14440.1 | IX |
| qHD2-1 | Seita.2G062000 | AT4G22140 | EBS | LOC_Os07g08880.1 | VII |
|  | Seita.2G035300 | AT5G48890 | LATE | LOC_Os07g05900.1 | VI |
|  | Seita.2G085600 | AT5G63470 | NF-YC4 | LOC_Os03g14669.1 | II |
|  | Seita.2G071200 | AT1G18950 | DDR4 | LOC_Os07g09580.1 | I |
| qHD2-2 | Seita.2G444300 | AT5G02810 | PRR7 | LOC_Os07g49460.4 | I |
| qHD3 | Seita.3G199300 | AT3G04030 | MYR2 | LOC_Os05g40960.1 | IV |
| qHD5-1 | Seita.5G096900 | AT1G68840 | TEM2 | LOC_Os01g04800.1 | V |
| qHD5-2 | Seita.5G177000 | AT4G10180 | DET | LOC_Os01g01484.2 | VII |
|  | Seita.5G178100 | AT3G54500 | LNK2 | LOC_Os01g31360.3 | VIII |
|  | Seita.5G176700 | AT1G72390 | PHL | LOC_Os01g02860.1 | III |
| qHD6-1 | Seita.6G034500 | AT5G63470 | NF-YC4 | LOC_Os03g14669.1 | II |
| qHD9 | Seita.9G100700 | AT2G39810 | ESD6, HOS1 | LOC_Os03g52700.1 | VI |
|  | Seita.9G129400 | AT5G12840 | NF-YA1, HAP2B | LOC_Os03g48970.3 | II |
|  | Seita.9G113600 | AT1G09570 | PHYA | LOC_Os03g51030.3 | III |
|  | Seita.9G089700 | AT5G35840 | PHYC | LOC_Os03g54084.1 | III |
|  | Seita.9G119800 | AT1G68520 | BBX14 | LOC_Os03g50310.1 | I |
| qPL2 | Seita.2G444300 | AT5G02810 | PRR7 | LOC_Os07g49460.4 | I |
| qPL3 | Seita.3G199300 | AT3G04030 | MYR2 | LOC_Os05g40960.1 | IV |
| qPW1 | Seita.1G065300 | AT5G57660 | COL5 | LOC_Os06g44450.1 | I |
| qPW9-2 | Seita.9G468100 | AT5G63470 | NF-YC4 | LOC_Os03g14669.2 | II |

**TABLE S5** The information of millet varieties

| millet varieties | photoperiod sensitivity | Source |
| --- | --- | --- |
| Longgu3 | sensitive to photoperiod | Crop Research Institute, Gansu Academy of Agricultural Sciences |
| Longgu13 | sensitive to photoperiod | Crop Research Institute, Gansu Academy of Agricultural Sciences |
| Longgu25 | sensitive to photoperiod | Institute of Crop Resources, Heilongjiang Academy of Agricultural Sciences |
| Nenxuan15 | sensitive to photoperiod | Qiqihar Branch of Heilongjiang Academy of Agricultural Sciences |
| Shanxihonggu | sensitive to photoperiod | Chifeng Institute of Agriculture and Animal Husbandry Science |
| Jigu24 | sensitive to photoperiod | Crop Research Institute, Shandong Academy of Agricultural Sciences |
| Longgu38 | sensitive to photoperiod | Institute of Crop Resources, Heilongjiang Academy of Agricultural Sciences |
| Nenxuan18 | sensitive to photoperiod | Qiqihar Branch of Heilongjiang Academy of Agricultural Sciences |
| Changnong45 | sensitive to photoperiod | Millet Research Institute of Shanxi Academy of Agricultural Sciences |
| Changnong35 | sensitive to photoperiod | Millet Research Institute of Shanxi Academy of Agricultural Sciences |
| Canggu3 | insensitive to photoperiod | Cangzhou Academy of Agriculture and Forestry Sciences |
| Yugu1 | insensitive to photoperiod | Anyang Academy of Agricultural Sciences, Henan Province |
| Jigu21 | insensitive to photoperiod | Crop Research Institute, Shandong Academy of Agricultural Sciences |
| Yugu18 | insensitive to photoperiod | Anyang Academy of Agricultural Sciences, Henan Province |
| Jigu22 | insensitive to photoperiod | Crop Research Institute, Shandong Academy of Agricultural Sciences |
| Zhonggu2 | insensitive to photoperiod | Institute of Crop Science, Chinese Academy of Agricultural Sciences |
| Zhonggu9 | insensitive to photoperiod | Institute of Crop Science, Chinese Academy of Agricultural Sciences |
| Jigu168 | insensitive to photoperiod | Millet Research Institute of Hebei Academy of Agriculture and Forestry Sciences |
| Jigu39 | insensitive to photoperiod | Millet Research Institute of Hebei Academy of Agriculture and Forestry Sciences |
| Yugu35 | insensitive to photoperiod | Anyang Academy of Agricultural Sciences, Henan Province |
